# xQTLatlas: a comprehensive resource for human cellular-resolution multi-omics genetic regulatory landscape

**DOI:** 10.1093/nar/gkae837

**Published:** 2024-10-01

**Authors:** Yuran Jia, Hongchao Dong, Linhao Li, Fang Wang, Liran Juan, Yadong Wang, Hongzhe Guo, Tianyi Zhao

**Affiliations:** Faculty of Computing, Harbin Institute of Technology, Harbin 150001, China; Faculty of Computing, Harbin Institute of Technology, Harbin 150001, China; School of Medicine and Health, Harbin Institute of Technology, Harbin 150001, China; Faculty of Computing, Harbin Institute of Technology, Harbin 150001, China; School of Life Science and Technology, Harbin Institute of Technology, Harbin 150001, China; School of Medicine and Health, Harbin Institute of Technology, Harbin 150001, China; Zhengzhou Research Institute, Harbin Institute of Technology, Harbin 450000, China; Faculty of Computing, Harbin Institute of Technology, Harbin 150001, China; Zhengzhou Research Institute, Harbin Institute of Technology, Harbin 450000, China; Faculty of Computing, Harbin Institute of Technology, Harbin 150001, China; School of Medicine and Health, Harbin Institute of Technology, Harbin 150001, China; Zhengzhou Research Institute, Harbin Institute of Technology, Harbin 450000, China

## Abstract

Understanding how genetic variants influence molecular phenotypes in different cellular contexts is crucial for elucidating the molecular and cellular mechanisms behind complex traits, which in turn has spurred significant advances in research into molecular quantitative trait locus (xQTL) at the cellular level. With the rapid proliferation of data, there is a critical need for a comprehensive and accessible platform to integrate this information. To meet this need, we developed xQTLatlas (http://www.hitxqtl.org.cn/), a database that provides a multi-omics genetic regulatory landscape at cellular resolution. xQTLatlas compiles xQTL summary statistics from 151 cell types and 339 cell states across 55 human tissues. It organizes these data into 20 xQTL types, based on four distinct discovery strategies, and spans 13 molecular phenotypes. Each entry in xQTLatlas is meticulously annotated with comprehensive metadata, including the origin of the tissue, cell type, cell state and the QTL discovery strategies utilized. Additionally, xQTLatlas features multiscale data exploration tools and a suite of interactive visualizations, facilitating in-depth analysis of cell-level xQTL. xQTLatlas provides a valuable resource for deepening our understanding of the impact of functional variants on molecular phenotypes in different cellular environments, thereby facilitating extensive research efforts.

## Graphical Abstract

**Graphical Abstract:**
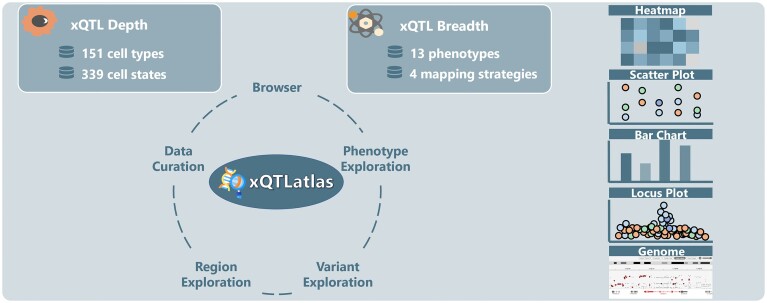

## Introduction

Genome-wide association studies (GWAS) have substantially enriched our understanding of genetic variants linked to human traits and diseases. Nevertheless, a predominant number of these variants are found in non-coding regions, complicating the elucidation of their roles and the molecular pathways they influence (1,2). The advancement of high-throughput technologies has significantly facilitated quantitative trait locus (xQTL) analysis, establishing connections between functional variants and molecular phenotypes at diverse biological levels (3,4), including gene expression (eQTL) (5), chromatin accessibility (caQTL) (6), histone modification (hQTL) (7) and DNA methylation (mQTL) (8). These resources enhance our comprehensive understanding of the genetic architecture, revealing causal relationships and functional impacts of genetic variants across multidimensional biological levels.

Large-scale xQTL studies such as GTEx (9), BLUEPRINT (10) and PsychENCODE (11) have advanced our understanding of the downstream causal effects of genetic variants. However, analyses based on averaged signals from tissue samples may obscure the specific cellular contexts in which functional variants influence their associated molecular pathways. Recent advancements in cell-resolution analysis have substantially enhanced our understanding of how genetic variations influence molecular phenotypes within specific cellular contexts (12–16). This progress reveals a complex interplay between genetic factors and cellular environments, crucial for unraveling the nuances of genetic regulation (17–21). For instance, Natri *et al.* utilized single-cell RNA sequencing (scRNA-seq) on lung tissues from pulmonary fibrosis patients and healthy controls, mapping eQTLs across 38 cell types, which revealed both shared and distinct cell-type-specific regulatory effects (22). Nathan *et al.* explored the impact of genetic variations in memory T cells, demonstrating how cellular dynamics, including cytotoxicity and regulatory capacity, significantly influence these variations (19). A pivotal direction for future genetic research is the precise characterization of the cellular contexts in which disease-associated variants modulate molecular phenotypes. This effort is essential for uncovering the molecular and cellular mechanisms contributing to disease susceptibility, deepening our understanding of the involved pathways, and facilitating the development of targeted therapeutic strategies (23–29). scQTLbase (30) and SingleQ (31) are pioneering databases specifically dedicated to housing eQTL research based on scRNA-seq. scQTLbase includes cellular-dependent eQTL (cd-eQTL) resources from 17 distinct studies, while SingleQ maintains a collection of 15 scRNA-seq resources. In addition to single-cell sequencing technology, there are other strategies for discovering cd-eQTL. These strategies include using purified cell type omics data to map cellular-dependent xQTL (cd-xQTL) for understanding genetic regulation in homogeneous cell populations (32,33), artificial intelligence models to detect cell type-specific regulatory effects (34), and cell deconvolution techniques to examine the interaction between genotype and cell abundance (35). Each of these strategies offers unique insights. However, current data resources do not comprehensively collect and organize cd-eQTL from these diverse approaches. Furthermore, the lack of cellular resolution genetic regulatory structure data at other molecular levels limits the depth and breadth of existing databases. These limitations highlight the urgent need for a comprehensive resource that not only systematically organizes cell resolution multi-omics genetic structures but also supports in-depth exploration and analysis.

To address these issues and meet the growing demand for a comprehensive and integrated cellular-level genetic regulatory landscape database, we propose xQTL Atlas (xQTLatlas). xQTLatlas integrates 796 datasets from 151 cell types and 339 cell states across 55 human tissues, covering 20 xQTL types. All cd-xQTL summary statistical datasets in xQTLatlas have been processed through standardized pipelines. These data enable researchers to systematically study the regulatory mechanisms of risk variants across multidimensional biological levels in various cellular contexts. xQTLatlas offers multi-level data exploration capabilities and unique interactive visualization tools, enabling cross-cell type, phenotype and genetic variants association analysis views. Additionally, xQTLatlas has significantly improved the user interface based on user-centered design principles, enhancing visibility and usability, and creating an environment conducive to intuitive data visualization and simplified queries. To our knowledge, xQTLatlas is the first comprehensive database to systematically organize multi-omics genetic structures of functional variants at the cellular level. We anticipate that xQTLatlas will become an invaluable resource for researchers, driving a deeper understanding of complex genetic regulatory mechanisms and promoting the development of precision medicine.

## Materials and methods

### Summary statistics collection and curation

**Figure 1.**
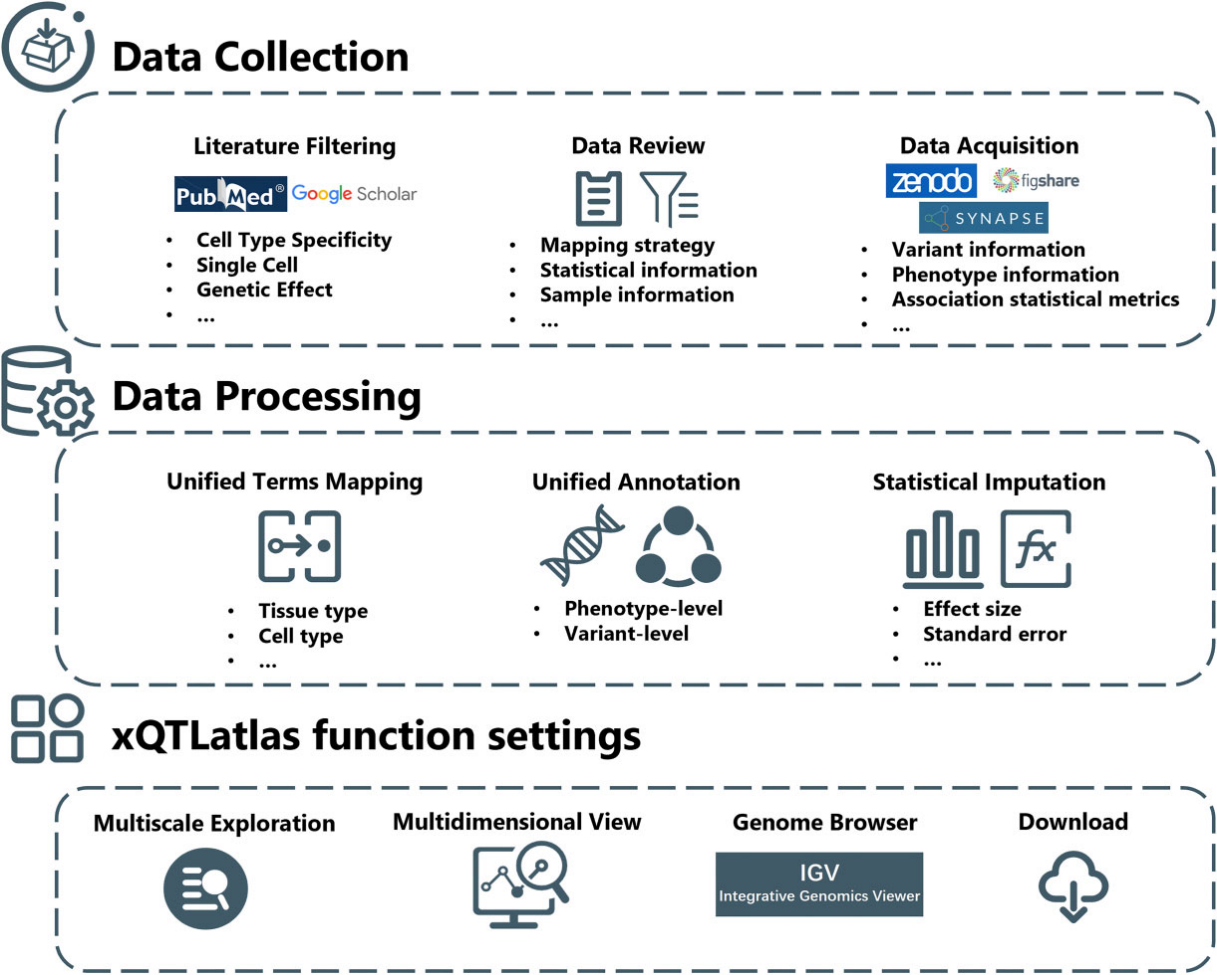
Overview of xQTLatlas. xQTLatlas pipeline begins with quality control using predefined publication filters, followed by a detailed examination and extraction of original variant-phenotype association data. Following this, statistical data are standardized and imputed, culminating in the development of functional modules for xQTL analysis and visualization.

In constructing our comprehensive atlas, xQTLatlas, we meticulously implemented rigorous filtering criteria to ensure comprehensive and accurate content (Figure 1). We began by conducting searches on NCBI PubMed and Google Scholar using predefined keywords and terms related to cell type specificity and xQTL analysis to identify relevant studies. We integrated datasets from various cd-xQTL mapping strategies including cell sorting, single-cell sequencing, interaction analysis and computational discoveries to maintain data integrity. We included studies featuring cells from diverse biological backgrounds—normal, treated, diseased or other stimulated conditions—and rigorously selected data from published research articles, excluding those lacking essential details such as critical information on variants, traits or statistical measures. Due to the limited availability and incomplete statistics of cell-level trans-xQTL data, trans-xQTLs are not included in the current version of xQTLatlas. All data are sourced from repositories such as Zenodo (https://zenodo.org/), FigShare (https://figshare.com/), GEO (36), Synapse (https://www.synapse.org/) and additional links provided in the original publications. xQTLatlas includes research from both genome-wide and regional cd-xQTL mapping, providing comprehensive coverage and ensuring that data can be traced back to the demographic information of the populations studied. We map these populations to categories defined in the 1000 Genomes Project (37)—AFR (African), AMR (Admixed American), EAS (East Asian), EUR (European) and SAS (South Asian), annotating datasets with multiple populations as ‘MIX.’ Given the variability of tissues, cell types/states and phenotypes in xQTL studies, we methodically categorized and annotated datasets from multiple sources. This methodical approach ensures that each cd-xQTL dataset can be precisely traced back to its original source, such as specific tissues, cell types or mapping strategies.

For all genetic variants, we systematically remapped the chromosomal locations from the original publications to the Genome Reference Consortium Human Build 38 (GRCh38). When the coordinates were documented in GRCh37, we employed LiftOver (38) to transition these to GRCh38 coordinates. Simultaneously, we updated the original dbSNP ID to the dbSNP156 release (39). Alleles for each cd-xQTL were preserved as documented from their original publications. The variety of cd-xQTL mapping strategies used across different studies led to significant differences in the format of summary statistics. We extracted key statistical metrics from each qualifying publication, including affected molecular phenotypes and their annotations, genetic variants and associated statistical measures. Drawing on established practices from existing databases (4,30), we standardized the format of cd-xQTL summary statistics within xQTLatlas. This standardization ensures that each entry consistently includes detailed information about phenotypes and variant locations, alleles, associated *P*-values, standard errors, effect sizes and false discovery rates. Where key statistical measures were absent in the original datasets, we supplemented these by imputing values based on available additional information. For example, in cases where standard error were not provided, we calculated them using available *P*-values and effect size.

To augment xQTLatlas, we have integrated systematic variant annotations from VannoPortal (40), providing detailed information on allele frequency, linkage disequilibrium, evolutionary conservation, functional evidence and variant pathogenicity predictions across various tissues and cell types. Furthermore, insights into potential causal relationships between risk variants and complex traits from CAUSALdb (41) have been incorporated to enhance the database’s comprehensiveness.

### Ontology standardization and categorization

xQTLatlas encompasses a wide array of molecular phenotypes, necessitating the standardization of terminology for both phenotypes and cell types to enhance the consistency and usability of the data. For each entry, we provide detailed annotations, including the phenotype type, genomic regions and specific names. Each phenotype is meticulously defined with specific description and annotation standards. For example, methylation phenotypes are annotated using probe IDs and genomic regions, whereas transcription and gene phenotypes are standardized based on genomic positions and names, employing the latest GENCODE (42) Release 46 annotations from the GRCh38 version. Phenotypes lacking standardized annotations are described by chromosomal positions, such as ‘chr6:32472840–32473244′ for caQTL, and specific histone modifications like ‘H3K4me1(chr2:2131351–2132034)’ and ‘H3K27ac(chr6:32270482–32272947)’.

In terms of organizing cell type classification in xQTLatlas, we conducted a detailed manual review of each cell type as reported in the original studies. For each entry in xQTLatlas, we aligned and standardized annotations according to the tissue source, cell type, cell state and cd-xQTL mapping strategies provided in the original research. The annotations of cell states include specific features described in the publications, such as particular gene states or conditions like ‘ATF1+’ for active transcription factor presence or ‘interferon stimulation 24 h’ to indicate cellular response to treatment. This systematic review and annotation process ensures that each cell type in xQTLatlas is accurately represented and reflects the detailed cellular context provided by the original studies. Cell types from studies that lack specific tissue source information and are derived from *in vitro* cultures are classified under ‘Other’. Moreover, we standardized terms from the original publications by mapping them to the naming conventions of the Human Cell Landscape (43) and GTEx (44), converting abbreviations to their full forms and aligning them with widely accepted reference names.

### Database design

xQTLatlas was constructed using a robust framework that incorporates MySQL (https://www.mysql.com) for database management and Java for backend development. Primary and composite indexing strategies were employed to optimize performance. A primary key index based on unique identifiers was used to expedite data retrieval and maintain data integrity. Additionally, a composite index, including chromosome and position columns, was implemented to accelerate specific queries. To further enhance performance, additional columns such as cell type, variant, phenotype and position were indexed, and a range-based partitioning strategy was adopted, segmenting the dataset into discrete, non-overlapping sections of 10 million bp each. The user interface of xQTLatlas was developed with HTML, Vue.js (https://vuejs.org/) and Plotly.js (https://github.com/plotly/plotly.js/), incorporating JavaScript libraries for enhanced interaction. Furthermore, IGV.js (45) was integrated for interactive genomic visualization. For optimal performance, we recommend accessing xQTLatlas using web browsers like Google Chrome or Microsoft Edge, ensuring efficient and effective use of its functionalities.

## Results

### Overview of xQTLatlas

The current version of xQTLatlas integrates summary statistics from 61 independent cd-xQTL studies, meticulously designed to ensure completeness and accuracy. Each dataset is manually annotated to include fundamental details such as tissue source, cell type and state. The entries in xQTLatlas are categorized into four main types based on the xQTL discovery methods detailed in the original publications: purified cell type xQTL, single cell xQTL, cell type/state interaction xQTL and *in silico* inferred cell type xQTL. These are further divided into 796 datasets affecting 13 molecular phenotypes (Supplementary Figure S1). Single-cell sequencing, which allow for a finer examination of cell heterogeneity and diversity within tissues, contribute the most extensive datasets, representing 46.8% of the total in xQTLatlas (Supplementary Figure S2). Additionally, studies employing purified cell type and single-cell sequencing for xQTL mapping are the most common sources of data, accounting for 56.5% and 25% of xQTLatlas, respectively (Supplementary Figure S3). xQTLatlas currently includes nearly 1.5 billion significant cd-xQTL entries (*P*-value < 0.05), covering 151 cell types and 339 cell states from 55 human tissues, providing an integrated genetic regulatory landscape with unmatched depth and breadth. xQTLatlas ensures accessibility through a user-friendly portal, enabling researchers without extensive computational backgrounds to engage effortlessly with the data. It features interactive modules for visualizing cd-xQTL summary statistics, including heatmaps, locus plots and scatter plots, along with a genome browser for visualizing genome-wide genetic associations. Moreover, all datasets and user-retrieved entries are downloadable in standardized formats for further analysis.

### Comprehensive data exploration module

**Figure 2.**
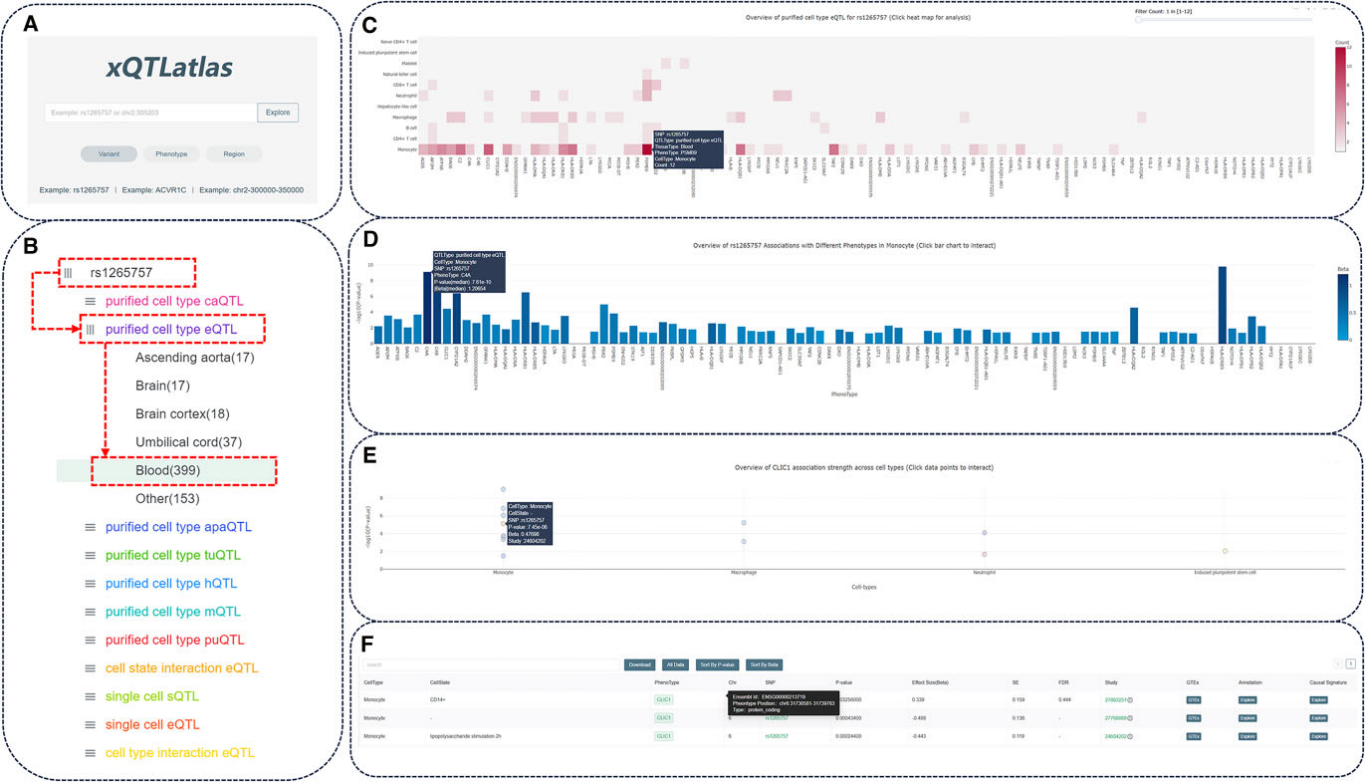
Demonstration of variant exploration in xQTLatlas. (**A**) The data exploration page of xQTAtlas supports three different modes. (**B**) The data overview interface after variant query is organized by xQTL type in the first level menu and by tissue in the second level menu. (**C**) Interactive heatmaps for data statistics. (**D**) Further statistical graphs based on xQTL statistics provide an overview of the association between different functional variants and phenotypes. (**E**) Comparison of the strength of the association between variant and phenotype in different cell types. (**F**) Summary statistics table.

xQTLatlas provides researchers with the capability to comprehensively explore cd-xQTL data from multiple dimensions. Using the data exploration interface (Figure 2A), users can efficiently delve into all functional variants and their associations with phenotypes, organized systematically by xQTL type (Figure 2B). xQTLatlas supports multi-level exploration across genetic variants, molecular phenotypes and regions of interest, enabling precise targeting.

***Exploring variant***. When searching for specific variants using their genomic position or dbSNP ID in variant search mode, users are initially presented with an overview organized by xQTL type and then by tissue type, as shown in Figure 2B. Users can then select a xQTL type of interest for further exploration. Upon choosing a specific subset of data, an interactive heatmap is displayed with the horizontal axis representing cell types and the vertical axis representing phenotypes. Each data cell in the heatmap represents a specific phenotype-variant association statistic, with the color intensity indicating the number of phenotype-variant associations (Figure 2C). Clicking on a data cell in the heatmap reveals the association strength and effect size for functional variants across different phenotypes within that cell type, where the height of the bars in the histogram is determined by the median −log10 *P*-value of the phenotype-variant associations, and the color represents the median effect size (Figure 2D). Additionally, the extent of specific phenotype-variant associations across cell types is also displayed, with data points colored according to the original publication (Figure 2E). Beneath the cross-cell type view at the bottom of the page, the summary statistics table is dynamically updated to reflect the user-selected data subset, simplifying the process of accessing and downloading relevant data (Figure 2F). Additionally, this dynamic table includes annotations of cellular context and phenotypes, as well as association *P*-values, effect size, standard error and false discovery rate for each xQTL. It also includes external links to the original publications and detailed information about the genetic variants. We have also integrated GTEx, VannoPortal and CAUSALDB, providing users with systematic and context-specific variant annotations and causal effects. This integrated approach not only enhances user interaction by providing tailored information but also facilitates the direct extraction of specific xQTL data from the interface, significantly boosting the utility of xQTLatlas for targeted genetic research.

**Figure 3.**
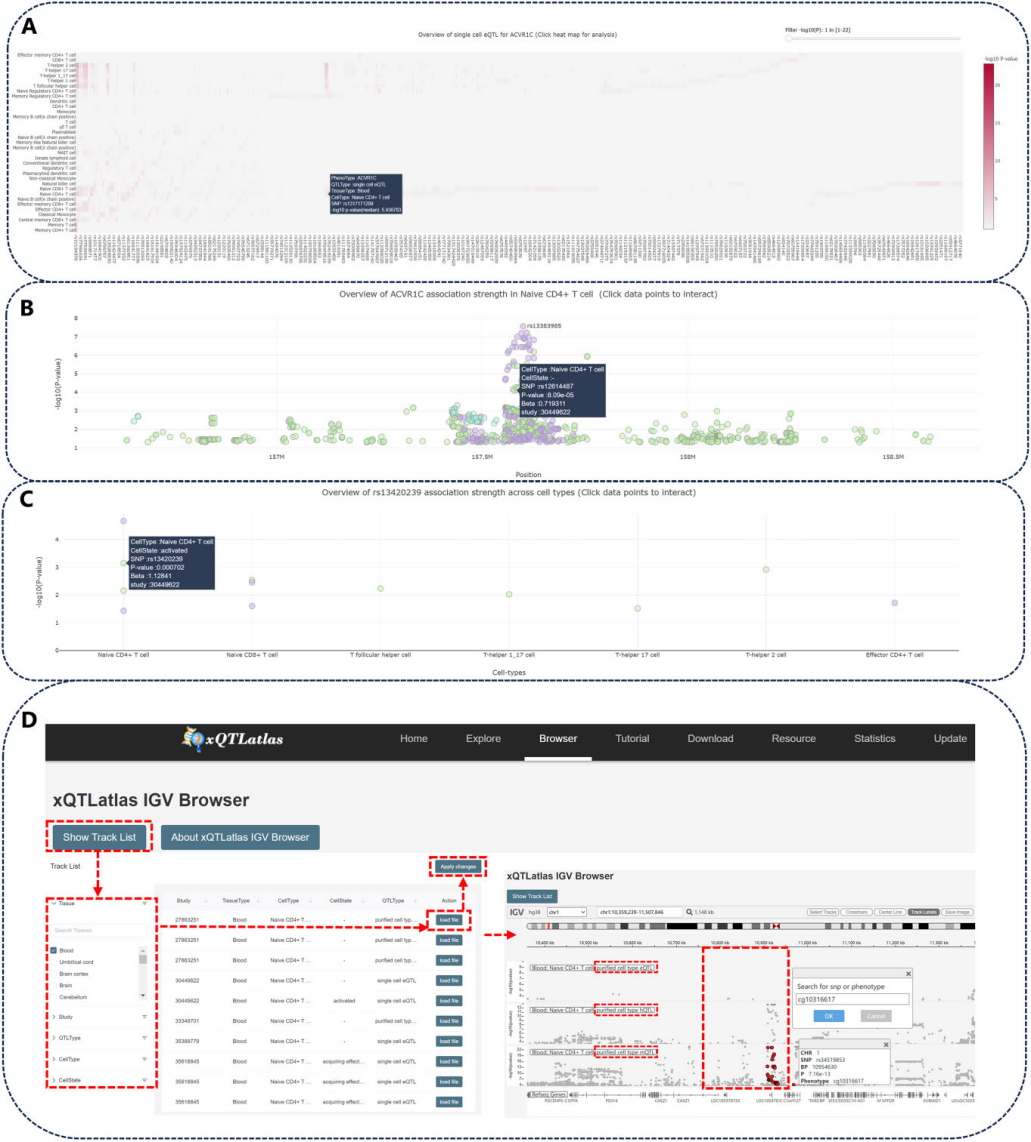
Demonstration of phenotype exploration and genome browser in xQTLatlas. (**A**) Interactive heatmaps for data statistics. (**B**) View of genetic regulatory structures for specific phenotypes. (**C**) View of phenotype-variant associations across cell types. (**D**) Genome browser of xQTLatlas visualizes the genetic architecture of different molecular phenotypes within the same genomic region in naive CD4 + T cell.

***Exploring phenotype***. In the phenotype search mode, users start with an overview similar to the variant mode. However, in the interactive heatmap, the vertical axis represents different functional variants rather than phenotypes, with each cell in the heatmap denoting specific phenotype-variant association statistics. The color intensity of each cell is determined by the median −log10 *P*-value (Figure 3A). Clicking on a heatmap cell displays a locus plot for the selected cell type, illustrating the distribution of functional variants associated with the phenotype. The most significant cd-xQTL are specially marked (Figure 3B), and the color of the data points is determined by the publication source. Below this, a visualization shows the cross-cell type associations of variants (Figure 3C), and all modules enable comprehensive interaction with the data points. Like in the variant mode, the summary statistics table below provides cd-xQTL information.

***Exploring regions of interest*.** Similar to the phenotype search mode, xQTLatlas enables customized exploration of the regulatory landscape within specific genomic regions. To ensure optimal performance, we recommend limiting the search range to within 200 kb. The interface features a search box in the overview section that allows users to query genetic associations for specific phenotypes within defined intervals. The data are organized in interactive heatmaps, locus plots and scatter plots, following the same user-friendly format as in the phenotype search mode (Supplementary Figure S4). This provides a consistent and efficient user experience.

### Genome-wide association strength browser module

xQTLatlas includes an interactive genomic browser that supports tailored exploration of diverse cd-xQTL datasets. Within the ‘Browser’ page, datasets are categorized by tissue, cell type, cell state, cd-xQTL mapping strategy and source of publication. Users can customize their analysis by adding multiple tracks to compare multi-omics cd-xQTL datasets. xQTL datasets of interest can be visualized by selecting them through the ‘Show Track List’ button (Figure 3D), and users have the capability to save the comparative genomic structure of a specific region as an SVG file. In addition, xQTLatlas supports precise localization of genomic regions, phenotypes or variants in tracks, and highlights specific cd-xQTL data to better target user needs. Clicking on data points reveals detailed information about each cd-xQTL, including the dbSNP ID and the associated *P*-value. For instance, xQTLatlas illustrates a visualization of the genetic variants impacts on gene expression, histone modification and methylation within the same genomic region in naive CD4 + T cells (Figure 3D). In this context, functional variants exert a more significant regulatory effect on molecular phenotypes at the levels of methylation and histone modifications than on gene expression. This indicates that epigenetic modifications play a predominant role in shaping the functional landscape of this region, potentially directing differential gene activity that could affect cellular behavior and responses. This underscores the complex interplay between different molecular mechanisms and emphasizes the utility of xQTLatlas in dissecting the multilayered genetic influences on cellular functions.

## Discussion and future directions

We have developed xQTLatlas, a comprehensive database designed to explore genetic regulatory landscapes at the cellular resolution. xQTLatlas supports multiscale data exploration and offers multidimensional visualization and analysis capabilities. It incorporates data from an array of cell types, cell states and cd-xQTL mapping strategies, enabling in-depth analysis to uncover the intricate regulatory structures of functional variants. xQTLatlas is distinguished by several innovative features: (i) The current version has meticulously curated hundreds of cd-xQTL datasets, organized in a hierarchical tree structure to facilitate efficient data access based on unique properties. (ii) Multi-level data exploration capabilities and distinctive interactive visualization modules allow users to explore genetic regulatory landscapes across various scales. (iii) A genetic structural landscape browser for visualizes the association structures of multi-omics quantitative trait locus across the genome-wide. For further guidance, refer to the Tutorial page on xQTLatlas.

Compared to other resources that encompass partial cellular-resolution xQTL data, xQTLatlas exhibits significant advantages. Specifically, QTLbase (4) focuses primarily on tissue-level data, consequently providing limited cellular resolution xQTL. Furthermore, databases like scQTLbase, ImmuneRegulation (46) and SingleQ lack diversity in their xQTL mapping strategies, cell types, molecular phenotypes and visualization techniques. xQTLatlas integrates an extensive range of genetic regulatory resources based on various mapping strategies and molecular phenotypes, enhancing both the depth and breadth of the data. Additionally, by standardizing cell type terminology and systematically annotating functional variants along with their potential causal relationships, xQTLatlas significantly advances its practical utility. xQTLatlas features advanced visualization and exploration tools that enable interactive, multi-scale exploration, comparison and analysis of xQTL data. Its user-friendly interface significantly enhances researchers’ capacity to perform comprehensive studies of the genetic regulatory landscape, surpassing the functionalities of existing databases. As our understanding of the complexity and heterogeneity of biological systems deepens, tools like xQTLatlas become increasingly essential. It significantly advances the exploration of genetic regulatory mechanisms at the cellular level, which is crucial for elucidating how genetic variants operate across various cellular contexts.

We plan to regularly update xQTLatlas, adding new data and features every 6 months to ensure our users have access to the latest and most relevant data in cd-xQTL research. With the evolution of new paradigms in population genetics, such as *in vitro* quantitative trait locus studies (47,48), we are positioned to explore the effects of genetic variants in rare cell types and unravel the molecular basis of common genetic diseases. Additionally, we aim to incorporate a broader range of cell phenotypes, including response, migration and stress states, to serve a wider research community. Future plans also include expanding our analysis pipelines and visualization methods to enhance the functionality of xQTLatlas.

## Supplementary Material

gkae837_Supplemental_File

## Data Availability

xQTLatlas is freely accessible to users with no registration needed at http://www.hitxqtl.org.cn/. All the datasets curated can be downloaded from xQTLatlas website for the purpose of research.
