# Supplementary material for: xQTLatlas: a comprehensive resource for human cellular-resolution multi-omics genetic regulatory landscape
Source: Nucleic Acids Res. 2024 Oct 1;53(D1):D1270–7. doi: 10.1093/nar/gkae837 (PMC11701524; doi:10.1093/nar/gkae837)
Supplement: gkae837_Supplemental_File [file gkae837_supplemental_file.pdf]

Supplementary information

xQTLatlas: a comprehensive resource for human cellular-resolution multi-omics genetic regulatory landscape

Supplementary Figures

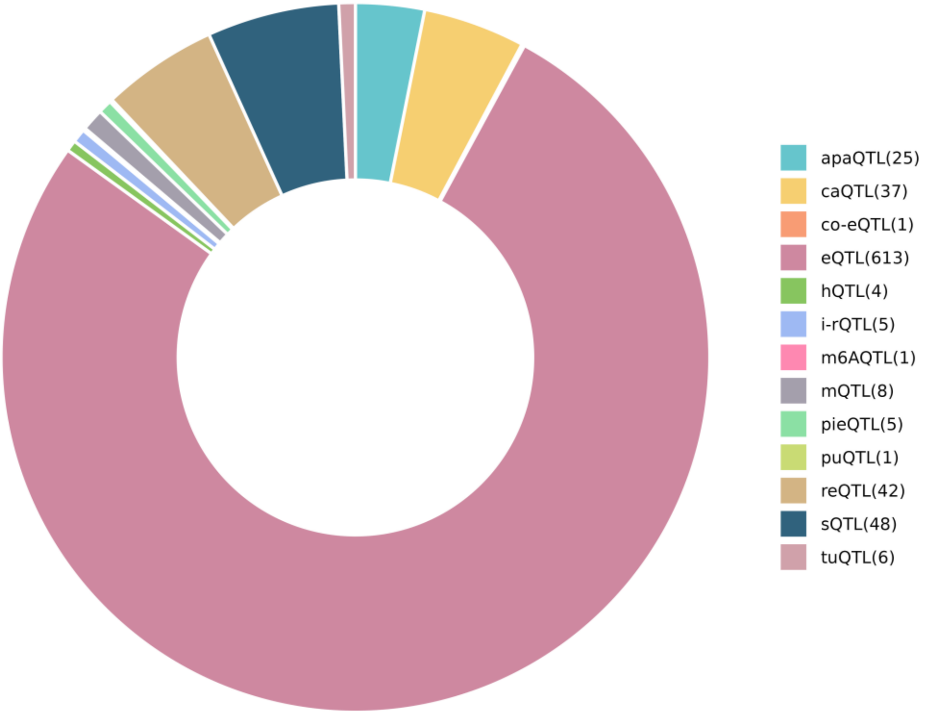

Supplementary Figure S1. Distribution of xQTLatlas Datasets by Molecular Phenotype.

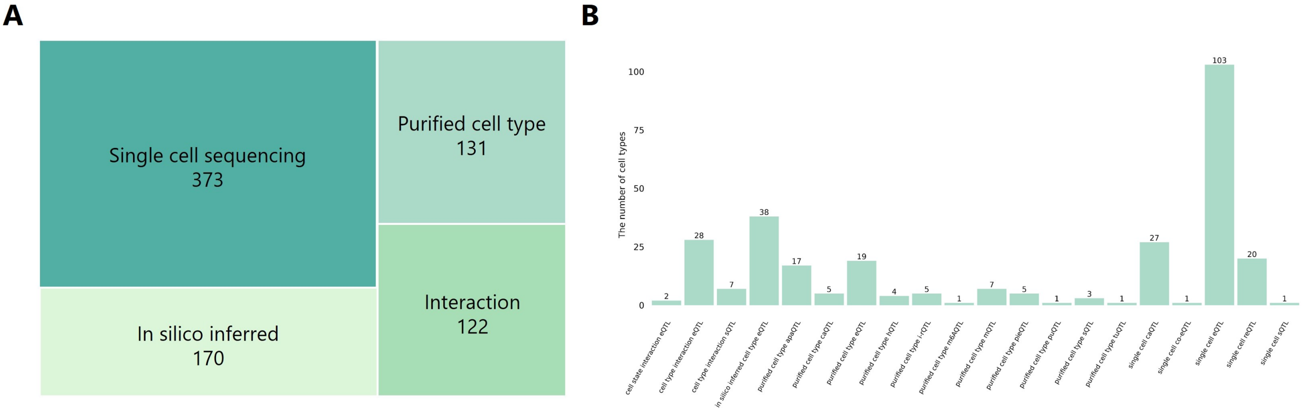

Supplementary Figure S2. The dataset distribution of xQTLatlas . (A) The Treemap presents the breakdown of different xQTL discovery strategy datasets in xQTLatlas. (B) The bar chart displays the number of cell types included in each xQTL type.

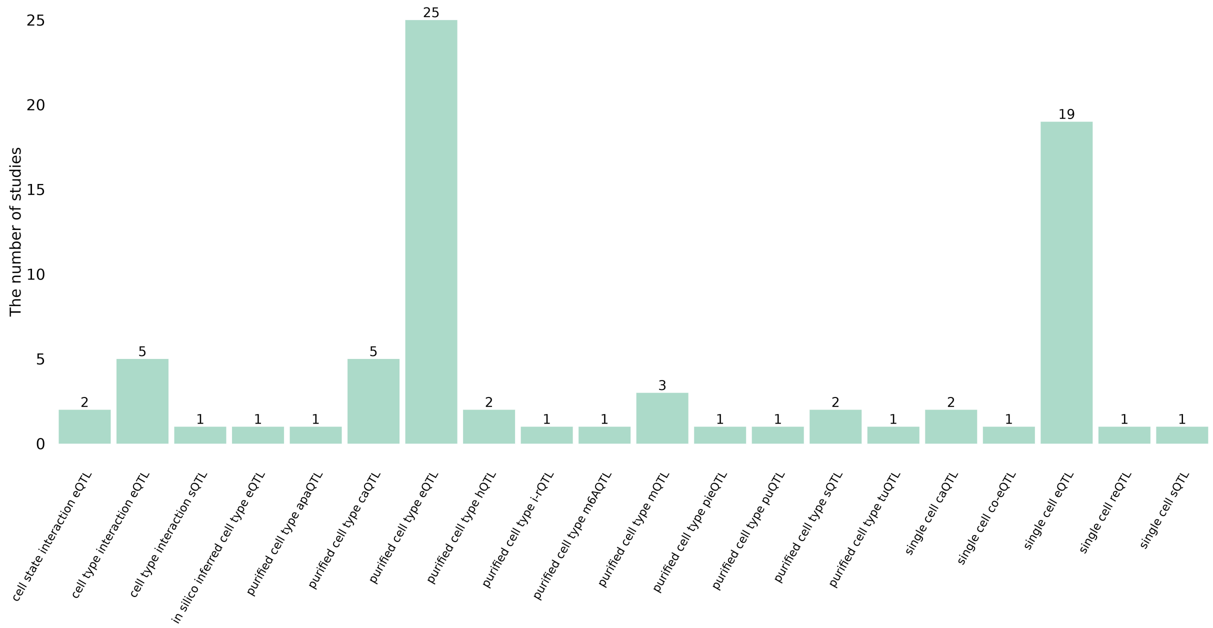

Supplementary Figure S3. The number of studies of different xQTL types in xQTLatlas.

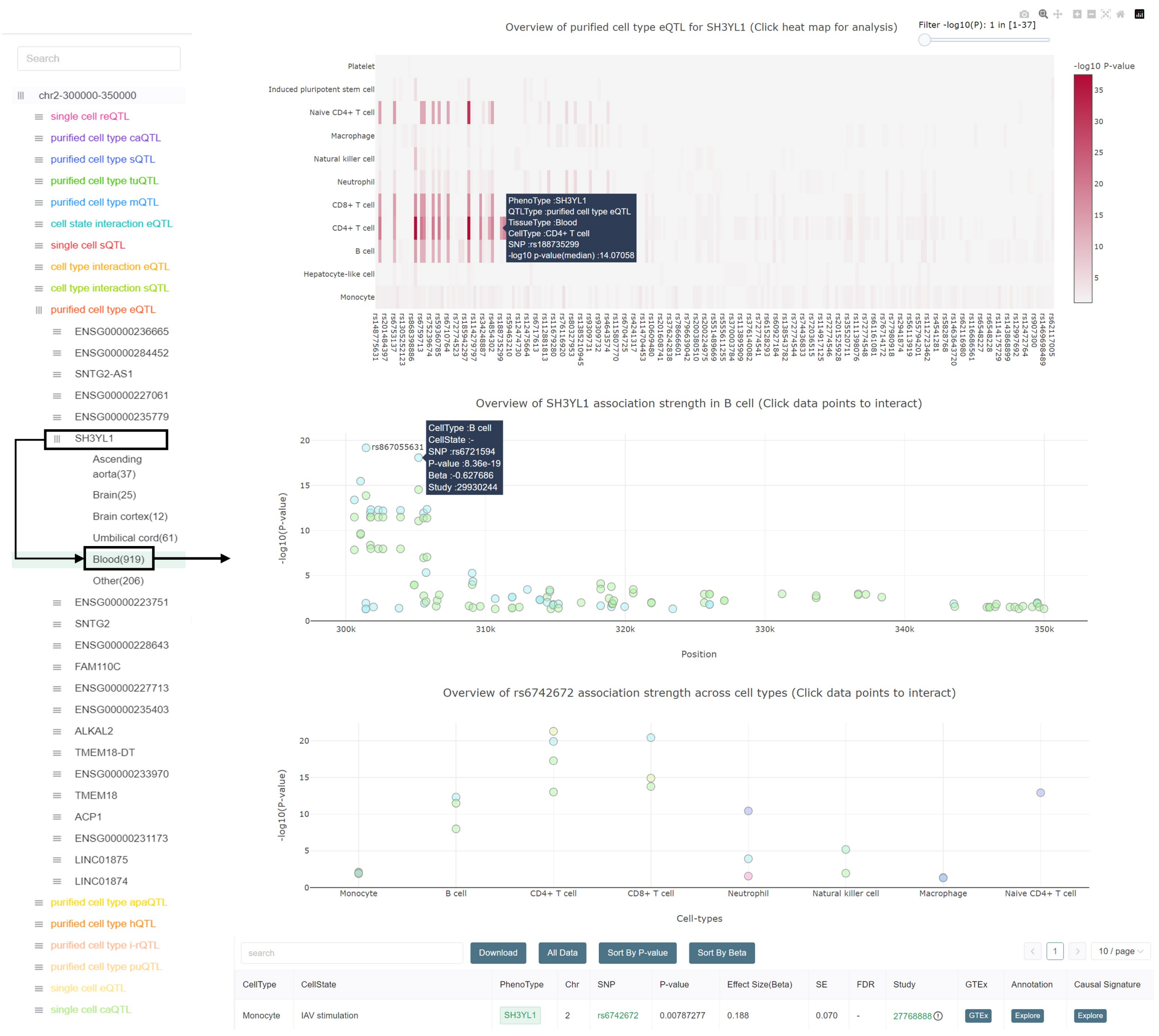

Supplementary Figure S4. Example of Region of Interest Exploration Mode.
